# A247 MEDITATION AND YOGA FOR IRRITABLE BOWEL SYNDROME (MY-IBS): A RANDOMIZED CONTROLLED TRIAL

**DOI:** 10.1093/jcag/gwab049.246

**Published:** 2022-02-21

**Authors:** A D’Silva, D Marshall, V Rajagopalan, Y Nasser, J Vallance, M Raman

**Affiliations:** 1 Medicine, University of Calgary, Calgary, AB, Canada; 2 Athabasca University, Athabasca, AB, Canada

## Abstract

**Background:**

When delivered in person, yoga is effective in managing Irritable Bowel Syndrome (IBS) symptoms. However, research is needed to determine the feasibility and effectiveness of yoga as a therapeutic option when delivered virtually.

**Aims:**

The aim was to explore the feasibility and effectiveness of a yoga program, delivered virtually, for patients with IBS. We hypothesized the program would be feasible and effective in improving outcomes in the intervention group compared to the control group.

**Methods:**

Adults diagnosed with IBS were randomized to receive either a Hatha yoga intervention or to an advice-only control group. The intervention consisted of facilitator-led weekly online classes for eight weeks delivered using Microsoft Office Teams and daily home practice. Yoga sessions included sequential delivery of postures, chanting, breathing exercises, and meditation over four weeks, with integrated practice over the final four weeks. Feasibility was evaluated with recruitment and attrition rates, adherence, and safety. The primary outcome was severity of IBS symptoms (IBS-Symptom Severity Score, IBS-SSS). Secondary outcomes included anxiety (General Anxiety Disorder-7), depression (Patient Health Questionnaire-9), and stress (Perceived Stress Scale) assessed at baseline and eight weeks. Unadjusted and adjusted analysis of variance (and covariance) models compared baseline and post-intervention data between groups using intent to treat analysis.

**Results:**

Sixty-five participants participated (32 treatment, 33 control). The mean age was 44.2±14.1 years and 91% identified as female. Participants had been living with IBS for 11.7±11 years. Fifteen participants were lost to follow-up (20% attrition rate). Participants attended on average 5.9±1.7 out of a possible 8 sessions (74% adherence) and accumulated 1,187±545 minutes in daily practice over eight weeks. No adverse events were reported. The groups did not differ at baseline (P>0.05). From baseline to post intervention, unadjusted ANOVA models indicated the yoga program was not statistically superior to the control group for IBS-symptoms (-17.5 points; 95% CI -62.6 to 27.6; P = 0.440), anxiety (-0.91 points; 95% CI -2.47 to 0.64; P = 0.245) and stress (-0.65 points; 95% CI -1.73 to 0.44; P = 0.239). Significant differences between groups were seen for depression (-1.82 points; 95% CI -3.49 to -0.15; P = 0.033). A second model considered relevant covariates including age, comorbidities, and years since diagnosis (i.e., ANCOVA), and the results were similar to the unadjusted model.

**Conclusions:**

Our virtual Hatha yoga and mediation program was feasible, and participants showed improvement in their depression scores. However, they did not experience a significant improvement in their IBS symptoms, anxiety, or stress, perhaps due to the short timeframe of the intervention.

**Funding Agencies:**

None

